# Large Eddy Simulation of Air Escape through a Hospital Isolation Room Single Hinged Doorway—Validation by Using Tracer Gases and Simulated Smoke Videos

**DOI:** 10.1371/journal.pone.0130667

**Published:** 2015-07-07

**Authors:** Pekka E. Saarinen, Petri Kalliomäki, Julian W. Tang, Hannu Koskela

**Affiliations:** 1 Finnish Institute of Occupational Health, Turku, Finland; 2 Leicester Royal Infirmary, University Hospitals Leicester, Leicester, United Kingdom; Technion—Israel Institute of Technology, ISRAEL

## Abstract

The use of hospital isolation rooms has increased considerably in recent years due to the worldwide outbreaks of various emerging infectious diseases. However, the passage of staff through isolation room doors is suspected to be a cause of containment failure, especially in case of hinged doors. It is therefore important to minimize inadvertent contaminant airflow leakage across the doorway during such movements. To this end, it is essential to investigate the behavior of such airflows, especially the overall volume of air that can potentially leak across the doorway during door-opening and human passage. Experimental measurements using full-scale mock-ups are expensive and labour intensive. A useful alternative approach is the application of Computational Fluid Dynamics (CFD) modelling using a time-resolved Large Eddy Simulation (LES) method. In this study simulated air flow patterns are qualitatively compared with experimental ones, and the simulated total volume of air that escapes is compared with the experimentally measured volume. It is shown that the LES method is able to reproduce, at room scale, the complex transient airflows generated during door-opening/closing motions and the passage of a human figure through the doorway between two rooms. This was a basic test case that was performed in an isothermal environment without ventilation. However, the advantage of the CFD approach is that the addition of ventilation airflows and a temperature difference between the rooms is, in principle, a relatively simple task. A standard method to observe flow structures is dosing smoke into the flow. In this paper we introduce graphical methods to simulate smoke experiments by LES, making it very easy to compare the CFD simulation to the experiments. The results demonstrate that the transient CFD simulation is a promising tool to compare different isolation room scenarios without the need to construct full-scale experimental models. The CFD model is able to reproduce the complex airflows and estimate the volume of air escaping as a function of time. In this test, the calculated migrated air volume in the CFD model differed by 20% from the experimental tracer gas measurements. In the case containing only a hinged door operation, without passage, the difference was only 10%.

## Introduction

The usage of hospital isolation rooms has been increasing world-wide after the demonstration of the airborne transmission potential of various infectious agents, such as severe acute respiratory syndrome-associated coronavirus (SARS-CoV) [1–6] and influenza viruses [7–11]—though for human-to-human airborne transmission of influenza, there has been an ongoing, fierce debate about the proportion of transmission that is truly airborne [12–18]. Concerns about airborne transmission have even been raised for Ebola virus during the current epidemic in West Africa, even though this virus is normally transmitted via direct contact [19]. It is this heightened awareness of the potential for airborne transmission of various infectious agents, together with the traditionally precautionary approach to infection control that means that patients infected with potentially highly contagious diseases are now being routinely quarantined in negative pressure isolation rooms to prevent further spreading of the disease, to protect patients, staff and visitors.

However, despite these precautions, containment failures can happen and the operation of the isolation room doors could be one of the main contributors [20–22]. A case study by Tang et al. [20] showed that the operation of isolation room doors can lead to containment failure. In the present study, the air migration from the isolation room induced by door-opening and passage has been modelled. Even though the air volume migrated (AVM) is closely connected to the number of pathogens potentially escaping, a linear dependence requires that the pathogens are aerosolized and fully airborne, and therefore capable of long-range transmission. Whilst this is the case for some infectious agents, others are transmitted by larger droplets, subject to gravitational settling. A recent review by Fernstrom and Goldblatt [23] summarizes the research on the aerobiology of infectious aerosols. The division between airborne and droplet transmission is complicated by the fact that in dry air, the droplets desiccate rapidly, leaving so-called droplet nuclei that are airborne. This may happen prior to the droplet/droplet nuclei reaching the floor. Or the droplet nuclei can be resuspended and become airborne again after settling onto a surface. The evaporation of water-containing droplets is significantly reduced in air of high humidity, making them more prone to settling under gravity [24]. According to the definition adopted by WHO, particles with a diameter of less than 5 μm are considered airborne. However, under normal air conditions, particles up to 50 μm dry out completely within 0.5 seconds, and particles up to 100 μm would totally evaporate before falling 2 m [25]. So droplets/particles of these diameters to begin with may quickly evaporate to fall within this so-called ‘airborne’ range. A human cough contains both droplets and fine, airborne particles that can be simulated by tracer gas [24]. The infectious droplet nuclei generated from human respiratory sources have a diameter 0.5–5 μm with average being less than 1 μm [26, 27]. The estimations about the extent to which airborne transmission contributes to the overall infection rates in hospitals vary from 10% to 30% [23].

Hence, there is a need to understand the flow patterns caused by door motion and human (staff) passage through these isolation room doorways. In this paper we compare a Computational Fluid Dynamics (CFD) modelling approach using a time-resolved Large Eddy Simulation method (LES), to measurements obtained from a full-scale mock-up with identical geometry, to determine, whether CFD is an accurate and robust alternative method for understanding and characterising the flow behavior leading to possible containment failure. To this end we also introduce methods to produce CFD-simulated smoke videos that are easily comparable to those produced experimentally in the same experimental scenarios.

Most hospital isolation rooms use manually-operated or automatic hinged doors, though perhaps an increasing number are now using automatic sliding doors. Thus, for the experimental full-scale isolation room model both options were allowable, though the hinged door model has been previously presented [28]. This paper combines a LES simulation with measurements from the full-scale experimental mock-up with the same geometry.

The test case was a simple isothermal scenario without ventilation, but including single hinged-door-opening and closing and a nurse passing through the doorway. Thus, the flow interactions arising from the moving door and human, alone, are presented, without any interference from any other sources. Addition of other factors such as ventilation, temperature differences between the rooms, or negative room pressure (i.e. unbalanced ventilation) would be a relatively simple next step. Flow structures near the doorway of the mock-up were examined and recorded in some detail by dosing smoke in one room and videoing its spread into the other room. Techniques were developed to construct similar simulated smoke videos from the LES results, enabling direct comparisons with the experimental videos. The ultimate purpose was to test the capability of time-resolved LES method to reproduce the full-scale air flows present in a real isolation room. The simulation was run on a standard workstation.

There are a few earlier studies reporting LES modelling of contaminant transport caused by door movement and passage through the doorway. Choi and Edwards [29] simulated a walking manikin going through an open doorway (i.e. in the absence of a door). In this study, they use their own immersed solid method [30] to model a walking human. They modelled several scenarios, varying the walking speed, initial position of the manikin, and initial distribution of the contaminant in the two rooms. The contaminant in their study consisted of particles, affected by gravity. The numerical methods used to solve the transport equations were also developed by the authors. In a later paper the same authors [31] simulated a more complex geometry, where a human is walking from a contaminated room to a clean room through a vestibule and two hinged or sliding doors. This simulation also includes ventilation (with an exhaust in the vestibule and small gaps below the doors), human plume, and several successive passages through the doors, with a gaseous contaminant. A clear advantage of the immersed solid methods is that they do not require re-meshing between the time steps, thereby allowing use of a large computational mesh. A study by Shih et al (case B in [32]), applied dynamic meshing to model the effect of opening and closing of a sliding door, without passage, on the spread of CO_2_ contaminant from a human source inside a negative-pressure isolation room. Since dynamic meshing is computationally heavier than the immersed solid technique, the size of the mesh was less than 100 000 cells, though it was enough to model the CO_2_ isosurfaces.

Measurements of cross-doorway airflow behavior by other researchers also exist, but with differing geometries, door-opening-closing cycles, and other experimental conditions, making comparisons difficult. However, based on extensive measurements in a water tank model and in a full-scale house, Kiel and Wilson [33] found an analytical formula giving the air volume migration (AVM) due to the combined effect of door-pumping and buoyancy. Their results were based on short door hold-opening times of the order of 1 s. In the isothermal case, without buoyancy, their formula reduces to AVM = 2.3 m^2^s *U*_d_, where *U*_d_ is the velocity of the centre of the door. Tang et al. [22] have performed experiments by scaling down the present geometry to a water tank model, and using Reynolds number equivalent lengths and velocities. Smoke was then replaced by a food dye. They tested four different door constructions with results presented qualitatively using online videos. One of these scenarios can be compared to the present case.

When validating the simulations against the experimental measurements, the total AVM through the doorway was also compared. In the experiments, the air volume migrating from one room into the other was measured by tagging the air with tracer gases, and initially dosing different gases into different rooms at time zero (i.e. initial conditions). The amount of tracer escaping into the other room then gave the AVM.

Note that this study is a continuation of an earlier study simulating the door cycle without passage, see Saarinen et al. [34]. These CFD tests are a part of a larger research project [28, 35], containing measurements in the full-scale mock-up described above, in several different scenarios.

## Methods

### The Test Case

**Fig 1:**
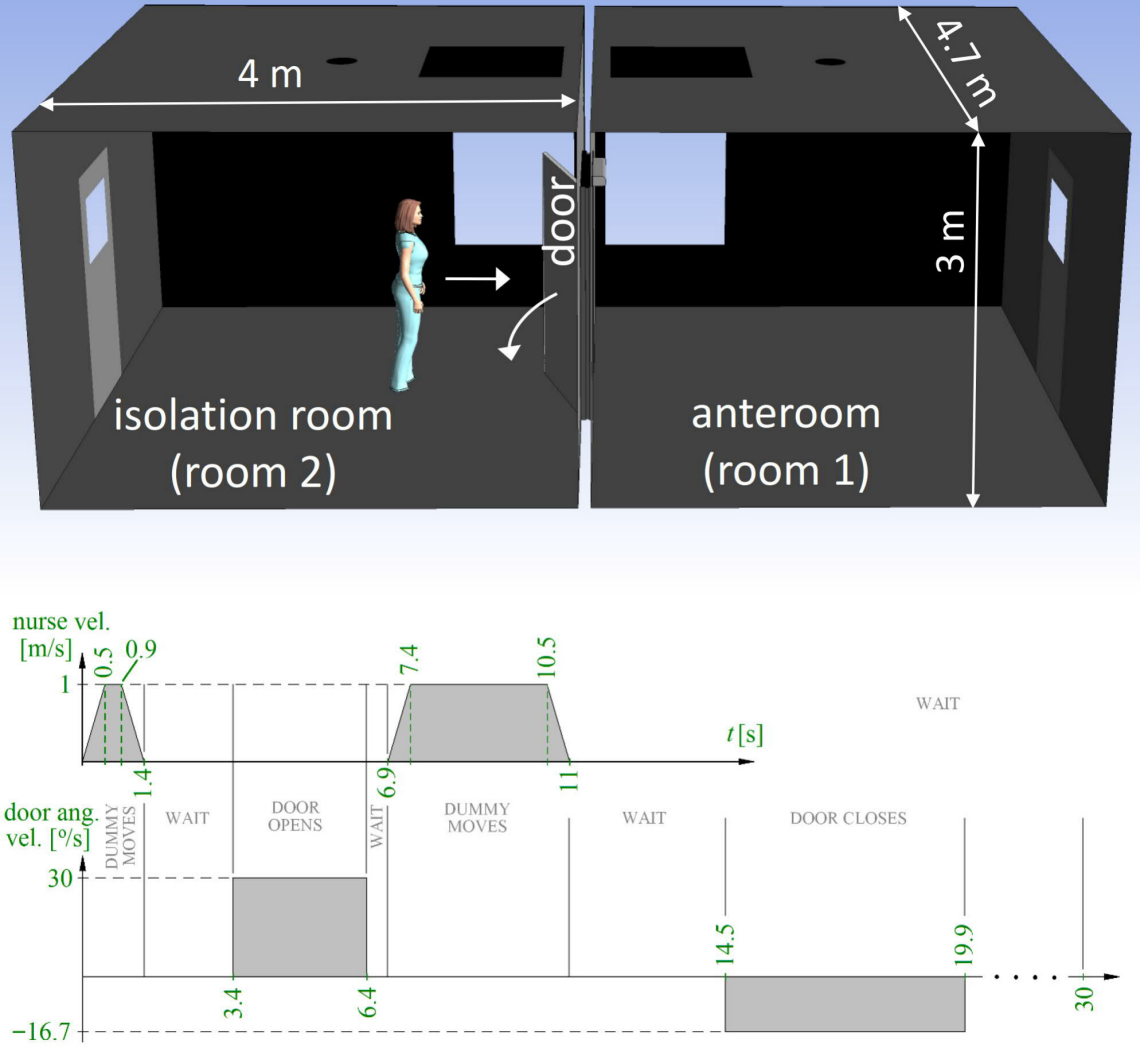
Timing of the test case. After an acceleration ramp, the manikin is moving with a steady velocity 1 m/s, ending in a deceleration ramp. The door moves with a steady angular velocity, different in the opening and closing phases.

The mock-up actually consists of two rooms, the isolation room being connected to an anteroom by a hinged door in the middle of a separating wall (Fig 1). The smallest width of the doorway is 1.10 m and smallest height 2.06 m. The (inner) width and length of both the rooms is 4.7 m and 4.0 m, their heights being 3.0 m, giving a volume of 56.4 m^3^. The volume of the doorway gap, including the thickness of the frame, is 0.23 m^3^, of which 0.09 m^3^ is covered by the door. A manikin standing on a wheeled cart, moving along a rail, is used to represent a nurse walking through the doorway. The cart and the door are both computer-controlled.

The cycle with door operation and passage, used in the CFD simulation, is detailed in Fig 1. The direction of the nurse movement is from the isolation room to the anteroom. There are four movement phases, separated by waiting periods. The movements are (durations in parentheses, see also Fig 1):

- Nurse approaches the door (1.4 s followed by 2 s wait).
- The door opens 90 degrees (3 s followed by 0.5 s wait).
- Nurse moves through the doorway, into the anteroom (4.1 s, followed by 3.5 s wait).
- The door closes (5.4 s).

After these movements, the simulation was continued for ten seconds to monitor the fading air flows. This makes the total duration of the test case 30 s. The door door-opening time used (3 s) was near the minimum, since the door operator was at almost full speed. The hold-open time in the test case was rather long (8.1 s) to make sure that the manikin would not collide with the door in the laboratory experiments. However, doors equipped with a door-pump or an automatic door operator may have longer cycle times.

Both in the experiments and in the simulation, the steady travel speed of the nurse was 1 m/s, preceded and succeeded by an acceleration or deceleration ramp of 2 m/s^2^. In contrast, the acceleration and deceleration of the door was so rapid that in the simulation it was opening and closing with constant angular velocity, without any ramps. The full opening angle of the door was 90 degrees.

### Simulation Details

The simulation was performed with ANSYS CFX software using an incompressible, time-resolved large eddy simulation (LES) solver with a LES WALE subgrid model. The time step was chosen to be very short to ensure that the flow would normally travel no more than one mesh node spacing during one step, i.e., the maximal Courant number stayed below 1. The length of the time step was a constant 2 ms, until after 22.3 s of simulated time. Then after the door and nurse movements had ended, it was increased to 4 ms. The computational mesh was a pure tetra mesh comprising 11.7 million nodes (control volumes, corresponding to 68.7 million elements). In order to reduce the mesh size, a refined volume of approximately 6 m × (1–2.4) m × 2 m (greater width near the volume swept by the door), or some 20% of the overall volume of the rooms, was created in the path of the moving nurse and door. A dense mesh is inevitable in this volume not only to resolve the air currents, but also because the immersed solid method applied uses the underlying mesh to resolve the surfaces of the moving objects. In the refined volume, a typical mesh element diameter was of the order of 1 cm, and the volumes of the finite volumes were 1.5×10^−6^–2.5×10^−6^ m^3^. Away from the refined area, the volumes increased to 1.5×10^−3^–4×10^−3^ m^3^. The discretization schemes used were second order backward Euler (transient scheme) and central difference (advection scheme). The two rooms together formed a closed system surrounded by wall boundary condition, and in the initial state the relative pressure as well as all the velocity components were set to zero everywhere. A 2 cm high gap was left below the door to avoid convergence problems in the beginning of the door-opening phase. This detail differed from the mock-up, where this gap was sealed by a moving seal that was automatically lifted when the door started to move. The two tracer gases were modelled as passive scalars (CFX additional variables) and at zero time there was only one type of gas in one room.

The moving door and nurse were modelled by using immersed solid technique available in CFX, see [36, 30]. By using this method, no re-meshing is needed between the time steps. Instead, at each time step a check is made on whether the mesh elements lying in the trajectory of the moving object are located inside or outside of the object. If inside, the fluid element is forced to follow the course of the moving object. One should note that the immersed solid surface is not explicitly resolved, but its accuracy is dependent on the density of the underlying mesh.

The total CPU-time spent was approximately 94×10^6^ s in a workstation equipped with 2.8 GHz Intel Xeon v2 processors. Therefore, use of parallel run is inevitable. On the other hand, the time step length of 2 ms used here is unnecessarily short most of the time, since the maximal Courant number stayed well below 1, mostly. Thus, the solver run can be carried out in a reasonable time by using effective parallelization and by using run-time adjustment of the time step, based on the Courant number.

### Calculation of Air Volume Migration from Tracer Gas Measurements

The volume Δ*V*_*i*→*j*_ of air migrating from room *i* to room *j* during the door and passage cycle can be measured if we mark the air in room *i* prior to the cycle. A standard method to do this is to release suitable tracer gas in the air in room *i*. Let us assume it is spread evenly throughout the room to have a uniform mass concentration *ρ*. After the door and passage cycle, if the air in room *j* is mixed with a fan, and the mass concentration of the tracer gas there *after the cycle* is measured and multiplied by the room volume, the migrated mass Δ*m* of tracer gas is obtained. Then the volume of room *i* air that has escaped into room *j* is

$$\Delta V_{i\to j}=\frac{\Delta m}{\rho }.$$

Even in the absence of ventilation, the volume Δ*V*_*j*→*i*_ of air migrated into the other direction, from room *j* to room *i*, does not need to be the same. This asymmetry comes from the fact that when the nurse passes through the doorway, the volume of air displaced by her travels in the opposite direction. By using two different tracer gases simultaneously, each dosed into different rooms, it is possible to independently measure the volumes of air passing through the doorway in each direction. In addition, these volumes can be calculated even if in the initial state the rooms are contaminated by tracer gas from the other room. This, in turn, enables measuring several door and passage cycles in succession, without a need to flush the rooms.

Let tracer gas 1 be initially dosed into room 1 and tracer 2 into room 2, but let us allow some mixing having taken place during preceding cycles. Then the average mass concentrations of the tracers in the other rooms *prior to the cycle* are nonzero, i.e. *ρ*_1, room 2_,*ρ*_2, room 1_ ≠ 0. Conservation of mass gives the net mass of tracer *i escaped* from room *i* as

$$\Delta m_{i,\;\text{room}\;i}=-\Delta m_{i,\text{room}j}=\Delta m_{i}=\rho_{i,\text{room}\;i}\Delta V_{i\to j}-\rho_{i,\text{room}\;j}\Delta V_{j\to i}.$$

Note that both mass concentrations are measured prior to the cycle, but calculation of the net migrated tracer masses Δ*m*_*i*_ and Δ*m*_*j*_ requires measurements of the concentrations after the cycle also, to get the differences. Writing the corresponding equation for tracer *j* and solving the pair of equations for the migrated air volumes Δ*V* gives

$$\Delta V_{i\to j}=\frac{\rho_{j,\text{room}\;j}\Delta m_{i}+\rho_{i,\text{room}\;j}\Delta m_{j}}{\rho_{i,\text{room}\;i}\rho_{j,\text{room}\;j}-\rho_{i,\text{room}\;j}\rho_{j,\text{room}\;i}}.$$ (1)

The two tracer gases used in the measurements were N_2_O and SF_6_, having characteristic infrared absorption bands well apart from each other and from the strongest bands of H_2_O and CO_2_. Prior to their release into the rooms, they were diluted with clean air to prevent them from stratifying near the floor. The gases were initially dosed into different rooms, but mixing was taking place during repeated door and passage cycles, whereupon calculation of the AVMs from the measured concentrations was based on Eq (1). The concentrations of both the tracers in both the rooms were monitored by one gas analyzer (BK1302) taking the sample through sampling tubes that were perforated throughout their lengths. In addition, after the door had closed, the air in the rooms was mixed by fans. These actions were necessary to ensure the sample taken by the gas analyzer is representative of the overall conditions in the room. A third measure of confirmation was use of repeated measurements, as mentioned above. The door and passage cycle was repeated and measured 6 times (7 times in the case without passage). The results were scattered without a clear increasing or decreasing trend. To ensure proper flushing of the sampling tube, the gas analyzer took the sample from the exhaust tube of a more powerful pump. The photoacoustic sample cell of the analyzer was very small, with a volume of 3 cm^3^. It was flushed with a flow rate of 5 cm^3^/s, giving a time constant less than one second. The flush time used was much longer than that, since there was no need to sample during the door cycle.

### Calculation of Air Volume Migration from the Simulation

In CFD simulation initial mixing of the tracer gases is no concern, since in the initial state we are free to define *ρ*_1, room 2_,*ρ*_2, room 1_ = 0. Eq (1) then simplifies to

$$\Delta V_{i\to j}=\frac{\Delta m_{i}}{\rho_{i,\text{room}\;i}}.$$ (2)

The migrated mass Δ*m*_*i*_ of tracer *i* can be calculated at any instant of time *t* from the corresponding simulated time step by integrating the tracer mass concentration over the volume of the other room, i.e.:

$$\Delta m_{i}(t)=\underset{\text{room}\;j}{∭}\rho_{i}(r,t)\text{d}V.$$ (3)

Inserting into Eq (2) gives Δ*V*_*i*→*j*_ as a function of time. Thus, a LES simulation gives not only the total AVM, but its evolution in time as well. This enables us to examine, how rapidly the isolation room air is escaping at different stages of the door cycle. While real tracer gases are invisible, a time-resolved simulation provides a possibility to actually see the mixing of the airs from the two rooms. This is demonstrated in S1 Video. In the video, the mass concentration of one of the tracer gases is coded by colours. Pure isolation room air is red and pure anteroom air deep blue. Any other colour is a mixture, 50%-50%-mixture being white.

### Preparing Simulated Smoke Videos from a CFD LES Simulation

Since the tracer gases are invisible, they were replaced by smoke when visualizing the flow structures experimentally, in real-time. Smoke was then released into one of the rooms, where it stayed for a while, letting the temperature difference to vanish. The smoke was then neutrally buoyant. Since flow-driven transport phenomena dominate over diffusion, after the door-opening smoke was carried into the other room by the air flows in the same manner as the tracer gases. Similarly, simulated smoke would be a useful illustrative tool to simulate the flow structures predicted by LES. Moreover, comparison of simulated smoke videos and real camcorder shots would be an interesting method to qualitatively validate the CFD results. Similar propagation of smoke and tracer gas makes it possible to use the same passive scalar to represent them both in the LES simulation.

**Fig 2:**
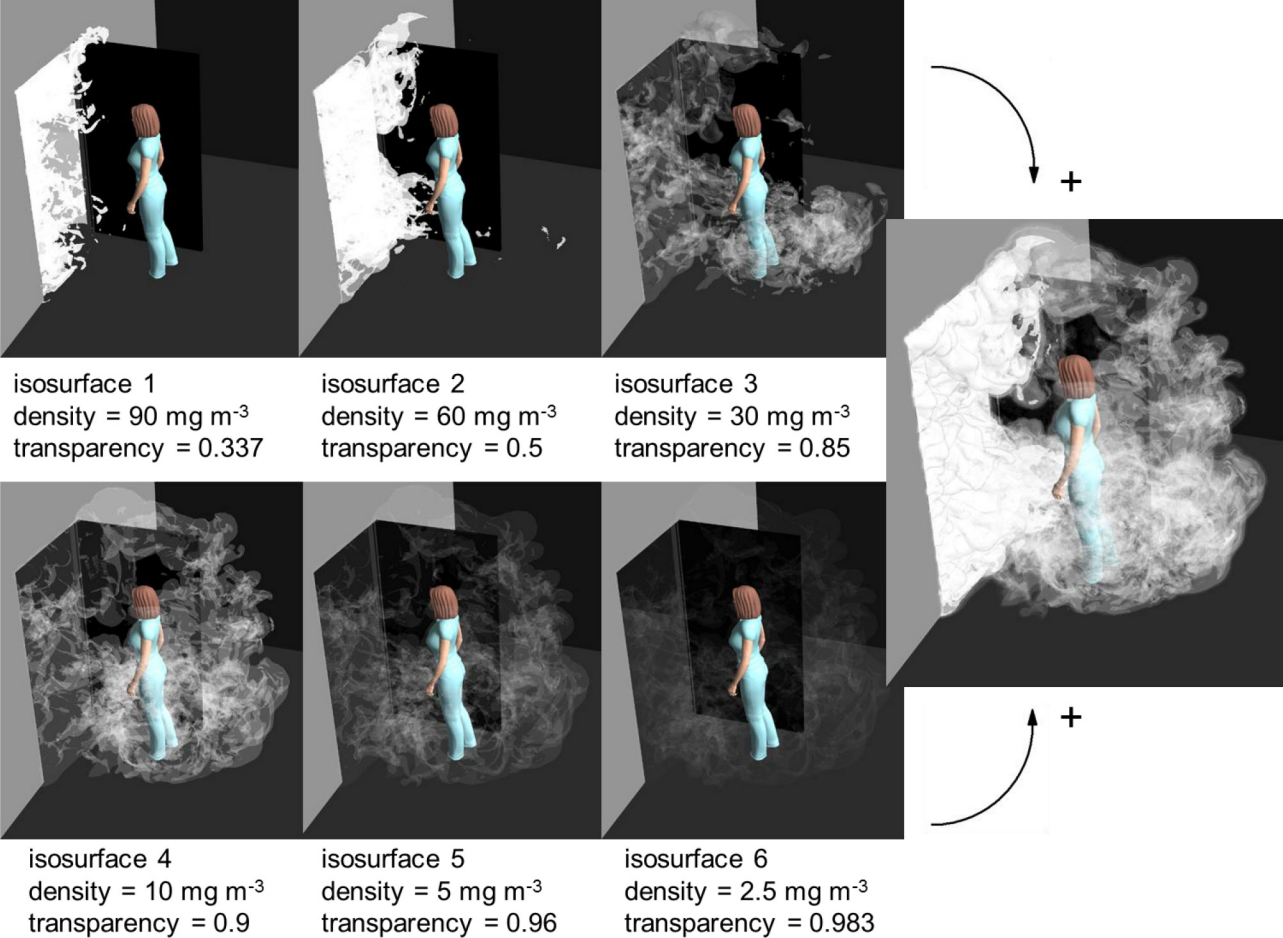
A generic method to prepare a simulated smoke visualization. Combining the six partially transparent isosurfaces of tracer gas concentration produces a simulated snapshot of a smoke experiment, as shown in the rightmost figure.

When penetrating deeper into the smoke, into areas of larger concentration of smoke droplets, the light scattering becomes more and more effective. This means that the smoke becomes less and less transparent. This effect can be discretized by first dividing the smoke into a few nested volumes with different concentrations. Next, let us approximate that all the light scattering is taking place on the limiting surfaces between these volumes. Then only a few isosurfaces of tracer mass concentration need to be drawn and a negative correlation between the concentration and the transparency of the surface needs to be defined. This principle is illustrated in Fig 2. On the two rows on the left, six isosurfaces with different tracer concentrations and transparencies are drawn. The colour of all the isosurfaces equals the colour of the light source, and is white. The rightmost drawing of Fig 2 displays the complete smoke simulation, with all the six surfaces drawn simultaneously. If the same is repeated at time steps separated by 0.04 s and they are shown with a frequency of 25 frames per second, we get a simulated smoke video in natural speed, easy to compare with an experimental one. An example is seen in S2 Video. It is a simulation combining two separate smoke experiments. Smoke escaping from the isolation room into the anteroom is coloured white, whereas smoke going the opposite way is coloured yellow, corresponding to a yellow light source. This video is based on a single CFD LES simulation, having different passive scalars dosed into each room in the initial state (corresponding to two tracer gases or two separate smoke experiments). Table 1 lists the isosurfaces and their transparencies, common to both passive scalars, used in preparing the video. There are no strict rules for the selection of these quantities. The only requirement is that different layers should be seen through each other in a natural-looking way. The video corresponds to situations where the rooms are entirely lit. It displays some basic flow structures, such as the jet through the gap below the door starting to open, the door vortices, and the wake of air the nurse is dragging behind her. It is comparable with the latter part of S3 Video by Tang et al. [22], showing the same features except that there is not a similar gap below the door.

**Table 1:** List of isosurfaces and their transparencies in the simulated smoke videos (S2 and S3 Videos).

surface	tracer gas mass conc. [kg/m^3^]	transparency
**isosurface 1**	60 × 10^−6^	0.5
**isosurface 2**	30 × 10^−6^	0.85
**isosurface 3**	10 × 10^−6^	0.9
**isosurface 4**	5 × 10^−6^	0.96
**isosurface 5**	2.5 × 10^−6^	0.983
**isosurface 6**	1 × 10^−6^	0.993

In the initial state the mass concentration of the tracer was 10^−4^ kg/m^3^ in the room it was dosed into.

**Fig 3:**
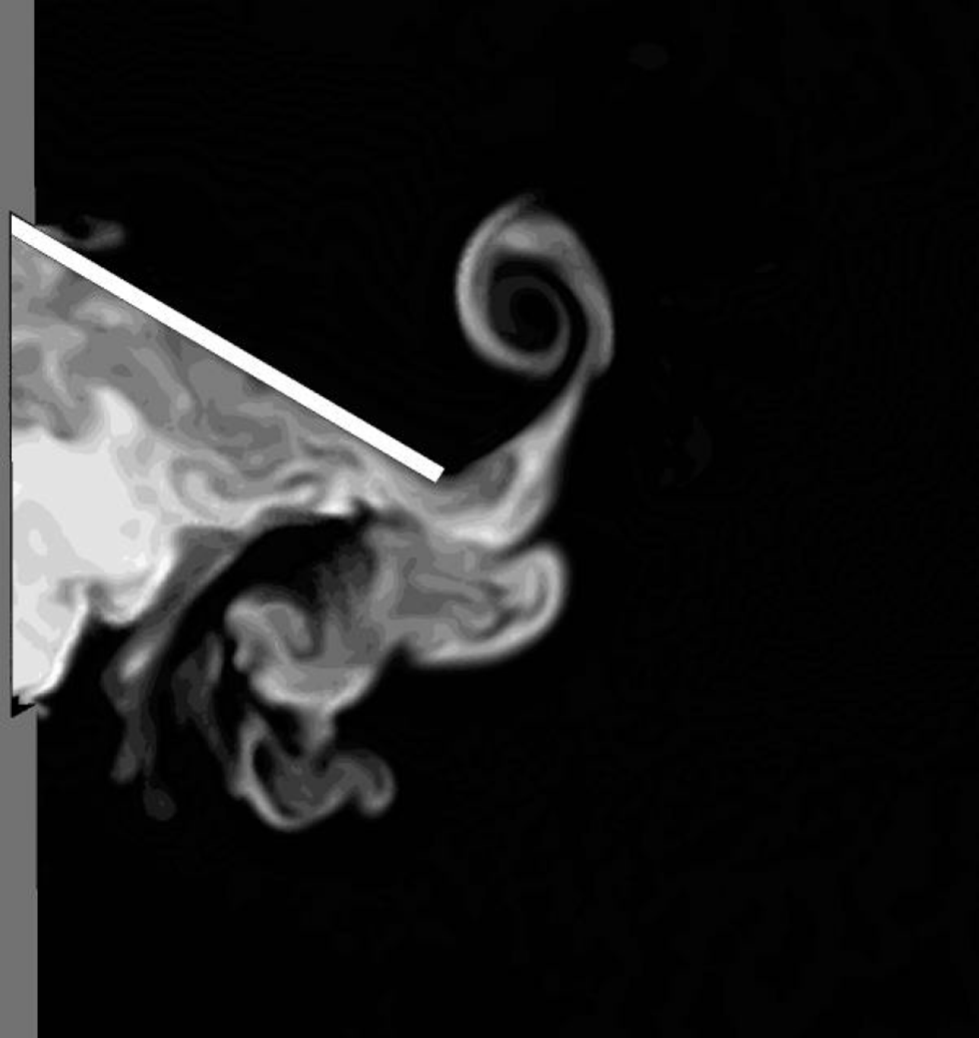
Simulation of smoke illuminated by a thin sheet of light. A greyscale contour map of the concentration of a passive scalar on a cross-sectional plane is equivalent to illuminating a thin sheet of smoke by white light.

Smoke is made visible by its ability to scatter light, necessitating a light source. Depending on the source, either a large volume, a strip, or only a narrow sheet of the smoke may be lit. When simulating the last-mentioned case, the isosurfaces reduce to isolines, since practically only a two-dimensional cross-section is lit. Then it is a better idea to plot a simple contour plot of the tracer (mass) concentration using a greyscale colour map. An example of this strategy is shown in Fig 3. It shows an instantaneous greyscale map of the tracer gas concentration near a closing door. The higher the tracer concentration, the whiter the hue, simulating the smaller transparency or the more intense scattering of light by smoke droplets. The door vortex is clearly visible behind the door.

**Fig 4:**
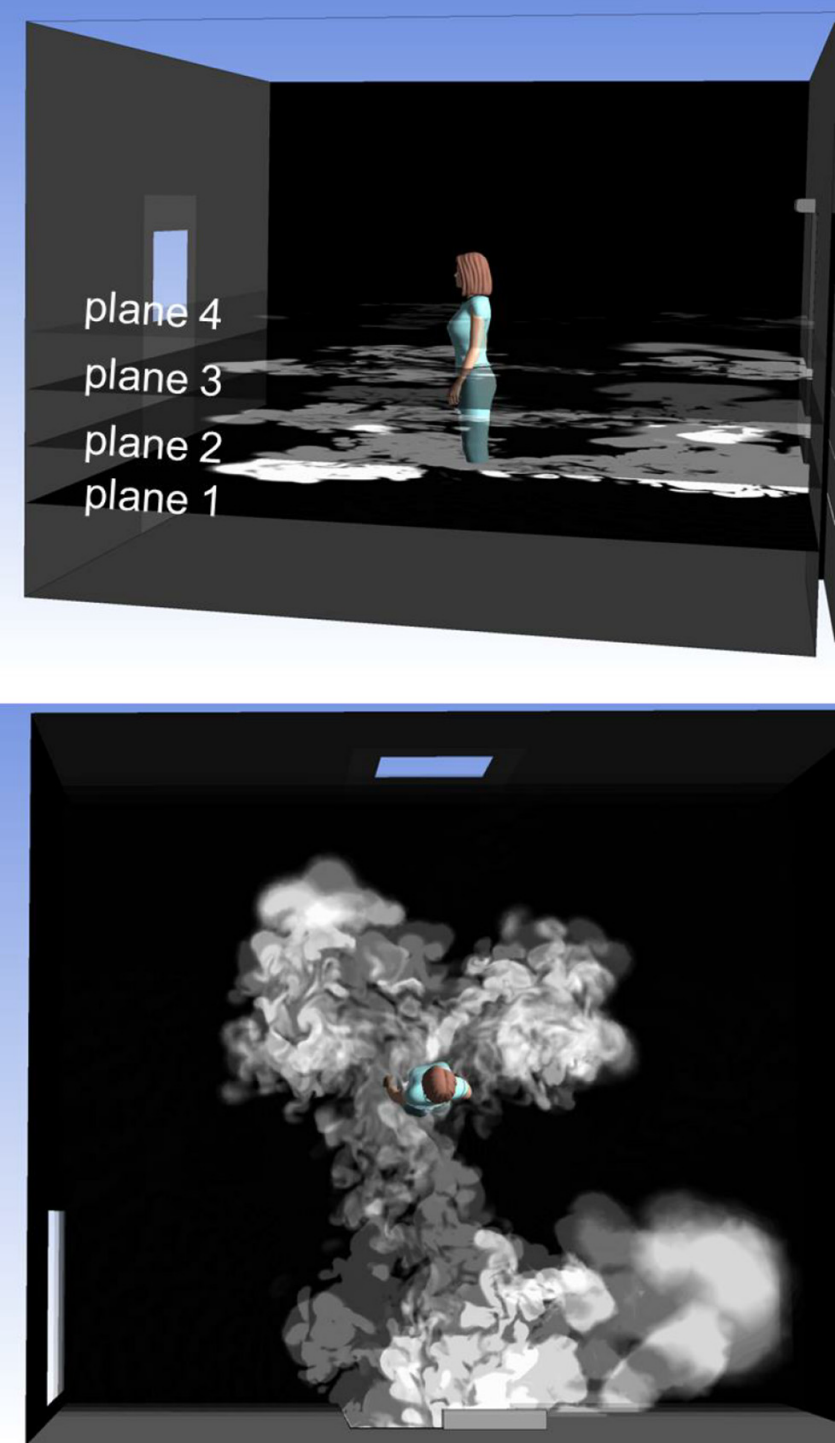
A simplified smoke simulation, viewable only from above. The upper figure shows four planar, greyscale contour maps of the tracer gas concentration at different heights. The lowest map is opaque, whereas the others are partially transparent. Together they simulate the smoke escaping from the isolation room, but only when viewed from above (lower figure).

The method of greyscale maps described above is also useful in a situation, where a thick zone of smoke is lit with light whose direction of arrival is approximately perpendicular to a coordinate axis. It is also assumed the view direction is approximately parallel to the same coordinate axis. Then the light scattering can be easily discretized in the view direction by drawing several partially transparent greyscale maps above each other. An example is presented in Fig 4. In the figure, four *xy*-oriented greyscale contour maps are drawn. Their transparency is increasing from bottom to top, so that when viewing from above, all the planes can be seen through each other. This creates an illusion of an illuminated horizontal thick zone of smoke. The method of contour maps is simpler and requires less computing than the method of isosurfaces, but is not suitable in a general case, where the line of sight is oblique.

## Results

### Comparison of Simulated and Experimental Air Volume Migration

The most important result from this study is the volume of air migrating from the isolation room to the anteroom, in this single-hinged-door with/without manikin passage scenario. This quantity can be easily calculated by Eq (1) from a tracer gas measurement, or by integrating the amount of tracer gas 2 in room 1 in a simulation, see Eqs (2) and (3). A LES simulation provides more detailed information than measurements here in that it gives the AVM as a function of time.

**Fig 5:**
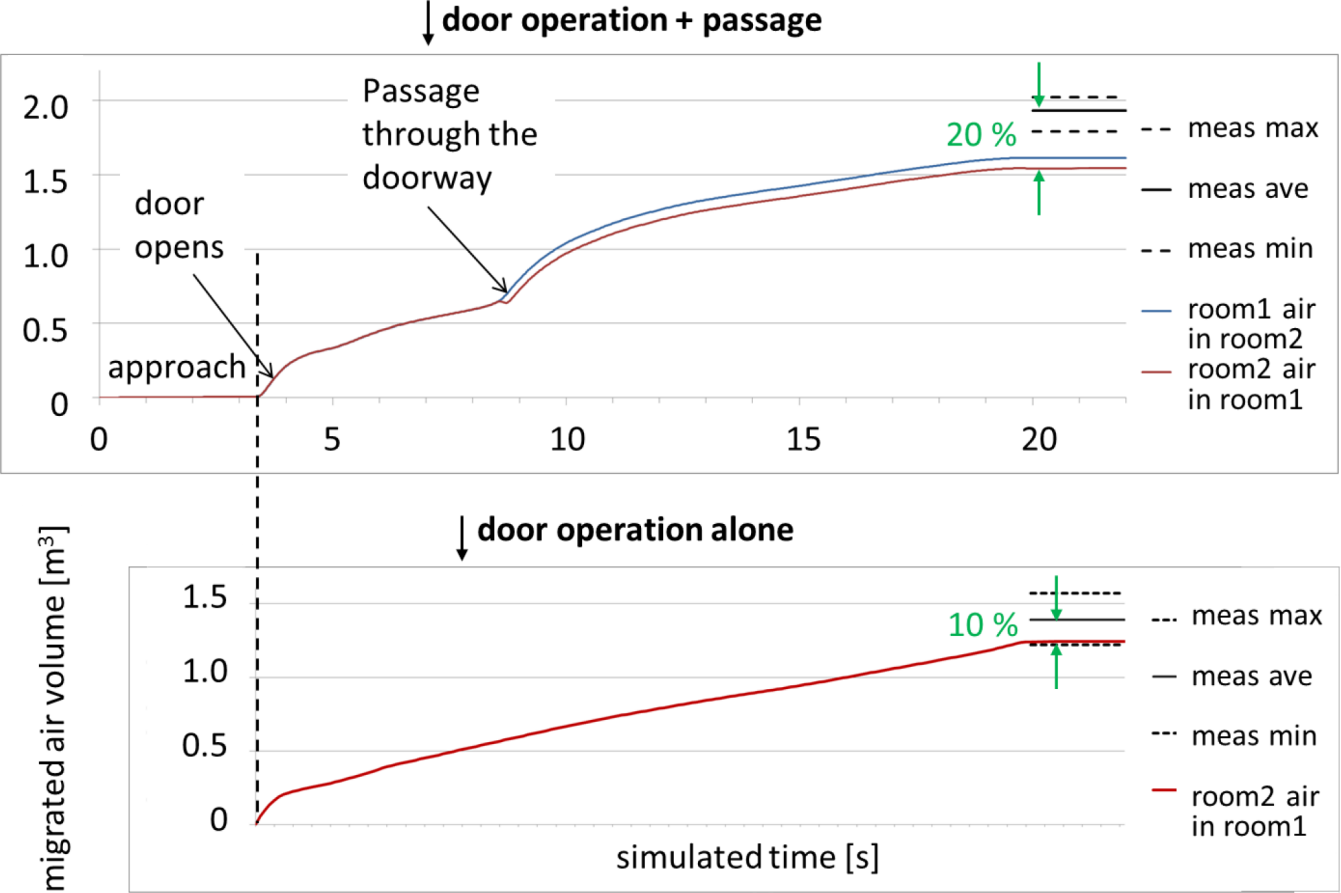
Air volume migrated: CFD LES compared to measurements. Time evolution of AVM as given by LES simulation during the door operation cycle with (top) and without (bottom) passage. The corresponding total values, as given by measurements, are also shown. The range of repetition measurements has been indicated by dashed lines.

The upper plot in Fig 5 shows the AVM(*t*) curve given by LES, together with the total AVMs obtained from a series of tracer gas measurements. A very intense flow through the doorway can be seen at the very beginning of the door-opening phase. This is due to the piston effect of the moving door, which in the beginning reduces the air volume in the isolation room. This effect causes a rapid counter-flow through the gap between the door and its frame. In the CFD simulation a 2 cm high gap was left below the door to avoid convergence problems. Another occasion of intense flow occurs when the nurse passes through the doorway. This is also the moment when the two AVM curves Δ*V*_1→2_(*t*) and Δ*V*_2→1_(*t*) separate, because the air displaced by the nurse moves from room 1 to room 2. The separation between the curves equals the volume of the nurse, which in this simulation was 0.070 m^3^ (generally, with a good precision, one litre per one kilogram of mass). Finally, the AVM curve of Fig 5 ends up at a value of 1.54 m^3^, 20% (or 0.39 m^3^) lower than the average of the repeated measurements at 1.93 m^3^. In another simulation, without the moving nurse, the corresponding result was 10% (or 0.15 m^3^) too low (1.24 m^3^ vs. 1.39 m^3^), see [34] and the lower plot of Fig 5. This plot also shows how the amount of migrated air is growing steadily during the waiting period when no object is moving and the door stays open. This is in accordance with our experiments, which revealed that the AVM grows steadily over an extended period of time during the hold-open phase, see Kalliomäki et al. [28, 35].

From the lower plot of Fig 5, the total simulated volume of air migrated in the door-opening phase (duration 3 s) is 0.412 m^3^. In the door-closing phase the flow continued at almost unchanged rate, the volume of air migrated being 0.327 m^3^. These volumes total to approximately 0.74 m^3^ by door-pumping alone, with the hold-open phase omitted. Including a hold-open time of 1 s, probably used by Kiel and Wilson [33], would increase the AVM to approximately 0.81 m^3.^ The analytical formula introduced by Kiel and Wilson would give AVM = 2.3 m^2^s *U*_d_ = 0.68 m^3^, which is rather near the simulated value. Here, one should note that in our case the door-closing time was longer (5.4 s) than the opening time (3 s), while they were obviously identical in the experiments of Kiel and Wilson. Since the flow rate stays almost constant during the closing phase, we can approximate that a closing time of 3 s would have resulted in air volume of 3/5.4*0.327 m^3^ = 0.182 m^3^ migrated during the door-closing. Then the total AVM would be 0.66 m^3^, which is almost exactly the same as the result by Kiel and Wilson. One should however note that the door used by Kiel and Wilson was an exterior door, not an interior door as in our case, and their data were rather scattered. In addition, Kiel and Wilson warn in their paper that their result for AVM due to door-pumping is not properly non-dimensionalized for the door size, and is therefore only valid for width *W* near 0.91 m used in their experiments. In our case *W* is 24% larger than this.

The difference between the final AVM with combined door operation and passage on the other hand, and door operation alone on the other hand, gives the additional effect of a human passage on the total AVM. It is not, of course, equivalent to the AVM due to human passage in the absence of the door operation. The value of this difference was 0.54 m^3^ in the measurements and 0.30 m^3^ in the simulations. Our measurements with a sliding door, with much less disturbance by the door itself, resulted in additional AVM of 0.92 m^3^–0.56 m^3^ = 0.36 m^3^ generated by the passage [28, 35]. This suggests that the human wake is an important agent in air transport. Concerning the relative proportion of the air volume of one room, migrating into the other room, Choi and Edwards [29] obtained a percentage of 2.5 in the case of walking speed 1 m/s and in the absence of the door. This means an AVM of 0.98 m^3^. This result was obtained 6 s after the human stopped, which makes it approximately comparable with the final AVM in our case. Also, the human stopped at 3.2 m from the doorway, while in the present study it stopped already at the distance of 2 m. Taking into account the deceleration ramp in our simulation, the time from the moment the nurse passes the doorway to the moment the door is half-closed is 8.45 s. In the paper of Choi and Edward, the time from the moment the human passes the doorway to the moment the AVM is measured, is 9.2 s. Thus, the time of influence of the wake is nearly the same in both simulations. However, their rooms and doorway were smaller than in our study (39.2 m^3^ against 56.4 m^3^ and 1.74 m^2^ against 2.23 m^2^). Tang et al. [21] estimated a range of values for the flux of the wake induced by a moving human, being 0.08–0.23 m^3^/s, though this estimate does not appear to take into consideration how the flux behaves over time during the passage and after the nurse has stopped moving.

According to the above, it is obvious that the AVM due to the human wake varies considerably depending on the geometrical details and on the details of the door operation. In case of a hinged door, the direction of the passage might also be crucial. In our case the nurse was moving against the movement of the opening door, and the effect of the passage was to reinforce the air migration. The situation might be different to the other direction [35]. Thus, one should be cautious to give a simple and generic rule of the effect of human wake on the AVM.

It is common in fluid dynamics to present results in a more general form by grouping two or more parameters into one or more non-dimensional variables. If we can state results by using such variables, they are applicable to any set of numerical values of the individual parameters. An obvious example is grouping flow velocity, duct diameter, and viscosity into Reynolds number. Since a large set of data with varying parameter values is required to test such general relations, the results are presented here in terms of original dimensional variables, such as simulated time and migrated air volume in Fig 5. A set of varied door and passage cycles was tested in the experimental section of our study [35].

The purpose of this paper is to report a CFD LES simulation in one example case, to aid in assessing the accuracy of the CFD approach and to introduce the computer modeled, multi-layered, smoke videos. The door parameters varied in the laboratory experiments were opening-time, hold-open time, closing-time, and opening-angle. It was noted then that reducing the opening-speed (in the absence of ventilation) resulted in larger AVM, or that the effect of increased duration overrode the effect of faster door movement. Moreover, without passage the air escape through the doorway continued at nearly constant speed up to the longest hold-open time tested (25 s). A similar effect has been found by Hathway et al. [37] who studied doorway flows generated by a single hinged-door in a small-scale water tank model. The effect of opening angle (90° vs 45° tested) was smaller, but clearly detectable. By contrast, the effect of a 2°C temperature difference between the two rooms resulted in 41% increase in the AVM in the case used in the CFD simulation [35].

### Comparison of Experimental and Simulated Flow Patterns

**Fig 6:**
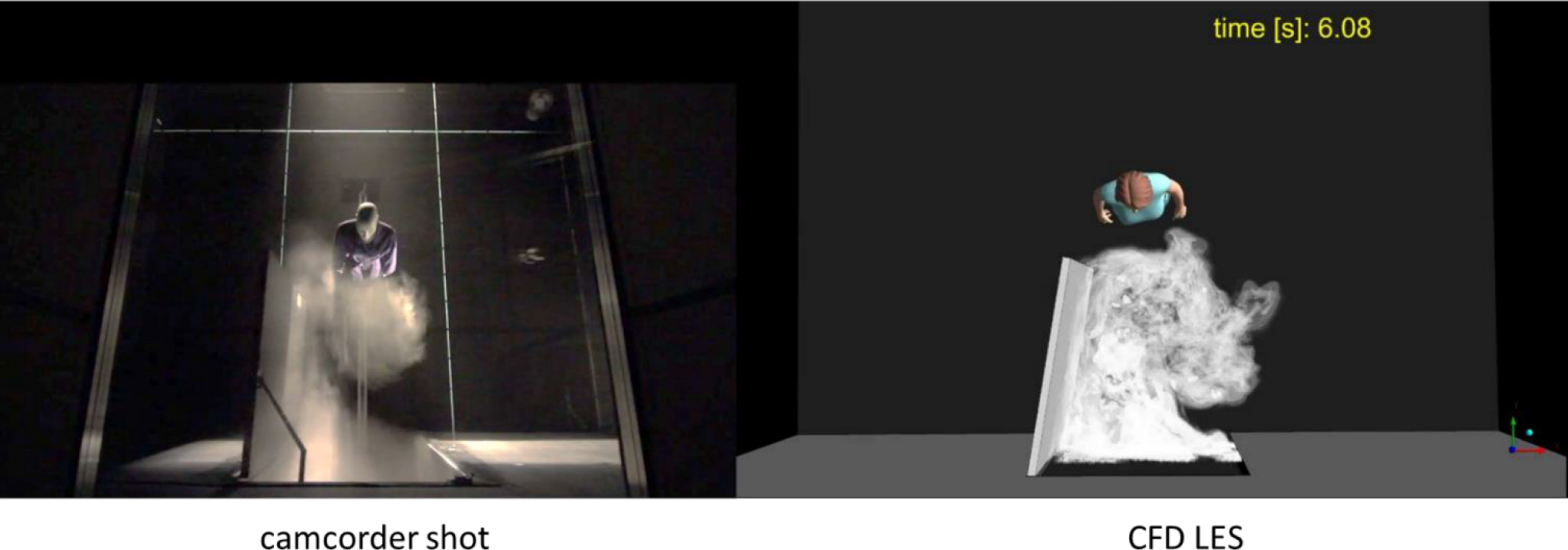
Smoke experiment in the isolation room side. Experimental (on the left) and simulated (on the right) smoke visualizations of the nurse exiting the isolation room. This figure is one frame of S3 Video, showing the flow structures as a function of time. Note that only a certain height interval is lit.

In order to visualize the air flows through the doorway, smoke was released in one of the rooms, and the smoke eddying into the other room was illuminated and videoed. The characteristic flow patterns near the doorway were found to be well discerned by illuminating a horizontal strip at the centre of the door, and shooting a video from above, through a ceiling window. Using the methods described above, corresponding simulated smoke videos were prepared and validated against camcorder shots. Fig 6 shows smoke travelling from the anteroom to the isolation room. Only the height interval from 0.6 m to 1.4 m above the floor is illuminated. The opening door vortex is just collinding with the nurse standing in front of the opening door. This figure is one frame from S3 Video, displaying the camcorder shot and the simulated (isosurface method) smoke as a function of time. Both the experiment and the simulation reveal the same principal flow features:

- An opening door vortex develops and separates from the door, as it swings open.
- After the nurse passage, a thick cloud of smoke pours through the door.
- A closing-door vortex develops behind the door, as it begins to close.
- After the door has closed, the stem of the vortex drifts to the right.
- The stem hits the side wall and continues along it.

The same phenomena can be seen in the water model experiments (see the latter part of S3 Video in [22]), but since the entire model is illuminated, the dye above the door blocks the sight to the closing door vortex.

**Fig 7:**
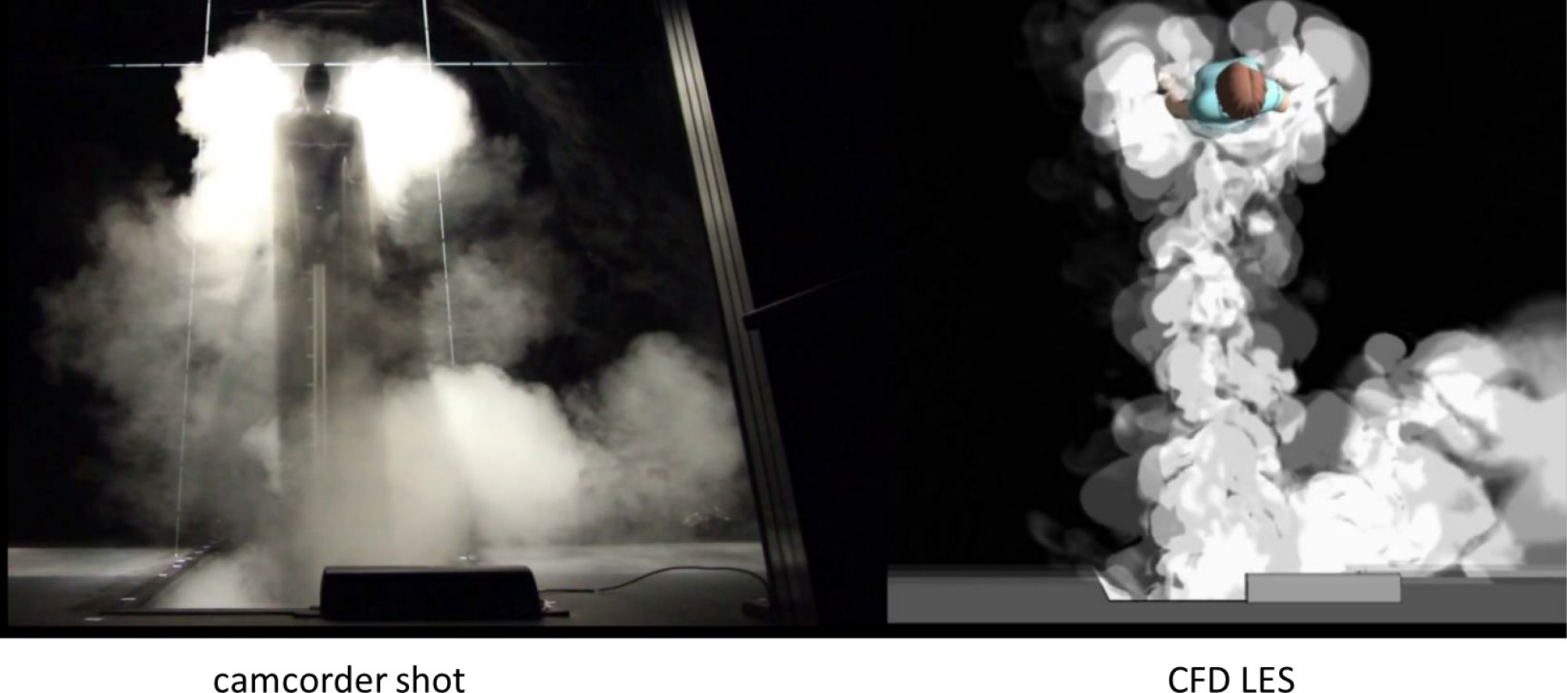
Smoke experiment in the anteroom side. Experimental smoke visualization as compared with simulated smoke drawn by the method of stacked contour maps. This figure is one frame of S4 Video. Again, a certain height interval alone is being lit.

In Fig 7 smoke has been dosed into the isolation room, and the anteroom side is shown. This time the simulated smoke has been generated by using the simplified method with four contour surfaces, as demonstrated in Fig 4. Again, the figure is one frame out of a time-resolved presentation, S4 Video. The main flow features, visible both in the experiment and in the simulation are:

- At the beginning of the door-opening motion, there is a rapid burst of smoke far ahead of the door.
- Soon after the door has started to open, the major air flow turns diagonally to the right.
- The nurse trails a wake of smoke behind her as she moves forward.
- When the nurse stops, the wake catches up and collides with her, splitting and passing to both sides around her, and starting to spread sideways.

Perhaps the most eye-catching difference is that in the experimental smoke video the opening door vortex extends further away from the doorway and is shooting more smoke on the left side of the room. This could be one reason for the difference between the measured and the modelled AVM. On the other hand, in the water model experiment by Tang et al. (the latter part of S3 Video in [22]), the left side of the anteroom is devoid of smoke during door operation and passage, just as in the simulation.

## Discussion

The simulated amount of air escaped from the isolation room was 20% smaller than the average in the tracer gas experiments. On the other hand, the simulated amount with a mere door cycle without the nurse/manikin passage was only 10% smaller than the corresponding experimental value. This suggests that simulation of the moving door and moving nurse both contribute a 10% relative error to the final simulated AVM. These were the first tests, and the results can probably be still improved by improving the accuracy of the computation and also of the geometry.

**Fig 8:**
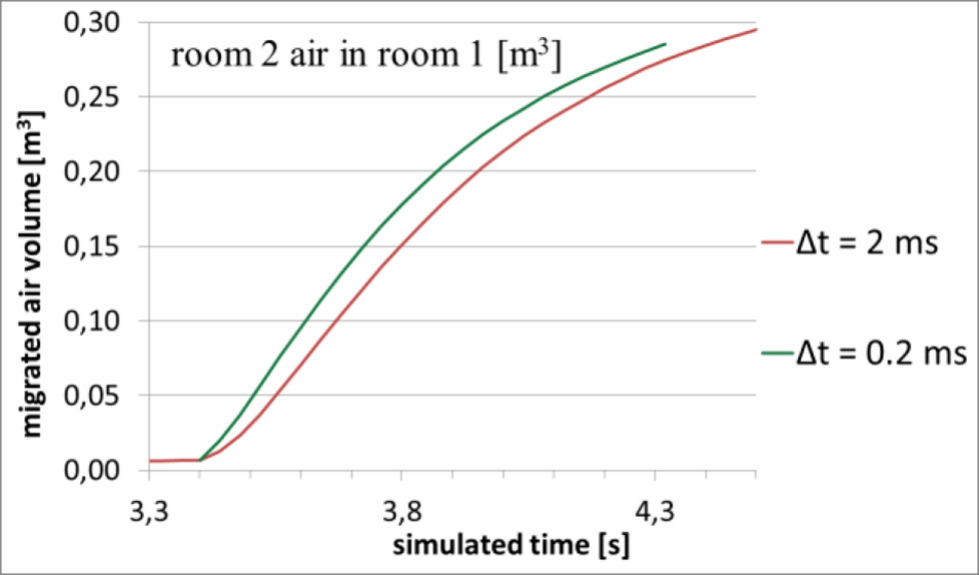
A test using a shorter time step. Green curve shows the result of shortening the time step by a factor of 10 at the beginning of door-opening. The AVM(*t*)-curve first rises 0.028 m^3^ above the original curve, but after that begins to approach it again.

The accuracy of the time-marching, when measured by the Courant number, was good. The maximal Courant number stayed well below one almost all the time. There were only two occasions of noteworthy excess of 1. The first occasion was just in the beginning of the door-opening, where the maximal Courant number momentarily rose to 5.68. Another occasion was the moment of door-closing, when the maximal Courant number reached the value 4.12. These values are not critical, and shortening the time step at these instances does not alter the results significantly, as shown by Fig 8. A recommended test of spatial resolution is to rerun the simulation by using a denser mesh. However, this was not practical due to the large computation time. Also, because of memory limitations, significant mesh refinement would not have been possible.

Part of the differences between experiments and simulation could be explained by inaccuracies in the modelled geometry. These include the gap below the door in the CFD simulation and differences in the real and simulated manikin. In the simulation, the under-door gap was present all the time, whereas in the experiments this gap was sealed until the door started to open. This discrepancy affects the pressure pulse generated when the door starts to push the air in the isolation room. This piston effect is not present in case of a sliding door, which is the next case we are modelling, thus enabling an estimate of it’s significance and impact of this effect. The nurse used in the simulation was a commercial CAD model of a female, not an exact model of the manikin used in the experimental measurements. The outfit worn by the CFD-modelled human was much more streamlined than the robe worn by the manikin in the experiments. Moreover, the wheeled cart under the manikin was not modelled.

In order to resolve the effects of door operation and passage, the basic case simulated in this paper did not contain ventilation. The effect of ventilation on the pathogen transmission is reviewed in the WHO guideline [25], based on 65 selected studies. It was obvious that the information about the impact of different types of ventilation on the reduction of infectious risks is insufficient. The main findings were that while insufficient ventilation increases the disease transmission and high ventilation rates could decrease it, there is no information that droplet-transmitted infections can be reduced by increasing ventilation rate. On the other hand, there is not enough data to define a minimum ventilation flow rate against infections through droplet nuclei (concluded also by Li et al [38]).

Concerning the ventilation strategies in isolation rooms, displacement ventilation is not recommended. The reason is that the large vertical temperature gradient characteristic to the displacement strategy can result in stratification of the exhaled air at some level [39, 24], and increase cross-transmission risks. Buoyancy also gives more time to the droplets to evaporate and become airborne.

A very large air flow rate without creating a draughty environment can be attained by using downward ventilation with large supply area [24]. Guidelines for isolation rooms do not recommend any particular ventilation strategy, but require keeping the room at negative pressure differential [40]. Kim and Augenbroe [41] suggest satisfying this by adaptive VAV (Variable Air Volume) operation, normally maintaining a low negative pressure differential, but temporarily inducing a higher value prior to door operation, to reduce energy consumption. As demonstrated by Adams et al [42], containment failure during door operation and passage can be significantly reduced by applying pressure difference. Even though the pressure differential immediately disappears when the door is opened, the supply and exhaust air flow differential, used to generate the underpressure in the isolation room, remains and works against the flow escaping from the isolation room.

In summary, if available, the use of modern workstations and parallel processing allows a time-resolved LES flow simulation to be a feasible method for room-scale flow modelling. The use of LES is attractive, since it enables the air flow features to be made visible, e.g. by using the simulated smoke method, as introduced in this study. Even though the present case was a simple, isothermal basic case without ventilation, the addition of ventilation would not be difficult and would not require much additional computing. In fact, this would be a natural continuation of this study. The effect of temperature differences between the rooms may be even greater than that of ventilation, and its effect could also be easily simulated. Finally, the thermal plume of the nurse (when not moving) has not been taken into account in this simulation, but this could also be simulated and should be taken into account in future models.

## Supporting Information

S1 Video Mixing of isolation room air and anteroom air. In this simulated video, featuring vertical cross-sectional plane in the middle of the rooms, (mass) concentration of one of the passive scalar variables is colour coded. In the initial state, isolation room (on the left) air is red and anteroom (on the right) air is deep blue. Any other colour is a mixture, equal distribution being white.
(MP4)

S2 Video A simulated smoke video combining two smoke experiments. This simulation is prepared by using the method of isosurfaces as detailed in the text. It combines two separate smoke experiments by using two passive scalars, originally dosed into different rooms. The smoke escaping from the isolation room is illuminated by white light, while the smoke going the other way (into the isolation room) is illuminated by yellow light.
(MP4)

S3 Video Experimental vs simulated smoke visualization as seen from above the isolation room. This combined video offers an opportunity to compare a real (left) and simulated (right) smoke video. In both videos only the height interval from 0.6 m to 1.4 m is lit to bring out the most interesting flow features.
(MP4)

S4 Video Experimental vs simulated smoke visualization as seen from above the anteroom. This combined video has been prepared by using the method of planar contour maps, and works only when viewed from above. Again, only the height interval from 0.6 m to 1.4 m is lit.
(MP4)
